# Complete remission of diffuse hepatocellular carcinoma in a young adult after GSP-TACE: a case report

**DOI:** 10.1186/1477-7819-12-300

**Published:** 2014-09-25

**Authors:** Song Liu, Yuewei Zhang, Guangsheng Zhao, Ying Liu

**Affiliations:** Department of Interventional Radiology, Affiliated Zhongshan Hospital of Dalian University, Dalian, P.R. China

**Keywords:** gelatin sponge particles, hepatocellular carcinoma, transcatheter arterial chemoembolization, young adults

## Abstract

Hepatocellular carcinoma (HCC) is the most common primary tumor of the liver. It mostly occurs in older age groups (usually those 50 to 60 years old), and rarely in young adults. The survival rate of these young HCC patients is usually very low. The authors report a case of a 22-year old man with diffuse-type HCC who successfully achieved complete remission for 46 months after second transcatheter arterial chemoembolization using gelatin sponge particles (Eric Kang Pharmaceutical Technology Co., Ltd. Hangzhou, China) combined with pirarubicin.

## Background

Hepatocellular carcinoma (HCC) is a common malignancy worldwide, especially in South East Asia and Africa [1] and is the most common primary tumor of the liver [2, 3]. Hepatocellular carcinoma can be classified into three types based on gross morphological characteristics: nodular, massive, or diffuse [4]. Although the nodular and massive types constitute the majority of HCC lesions, diffuse-type HCC still accounts for 7 to 13% of cases [5, 6]. Worldwide, HCC mostly occurs in older age groups (usually 50 to 60 years old), and rarely in young adults [7]. However, the age distribution of patients with HCC differs substantially in different parts of the world. In Africa, HCC often affects young people, with 40% of those afflicted being under the age of 30 [8]. In China, it is relatively rare for people under 35 years old to have HCC. The survival rate of these young HCC patients is usually very low [9]. Compared with adult HCC, morphologically the tumor is often massive, diffused, with quicker growth, stronger invasiveness, and poor prognosis [10]. Diffuse-type HCC almost always presents an advanced infiltrative tumor, so surgery is rarely indicated; rather, locoregional therapy in the form of transcatheter arterial chemoembolization (TACE) is generally the only available therapeutic option [11].

In this case, we performed TACE using gelatin sponge particles (GSPs) to treat a 22-year-old patient with diffuse-type hepatocellular carcinoma, and achieved complete remission. At the most recent follow-up, our patient had complete remission for 46 months after the second treatment, without any clinical disease recurrence.

## Case presentation

**Figure 1:**
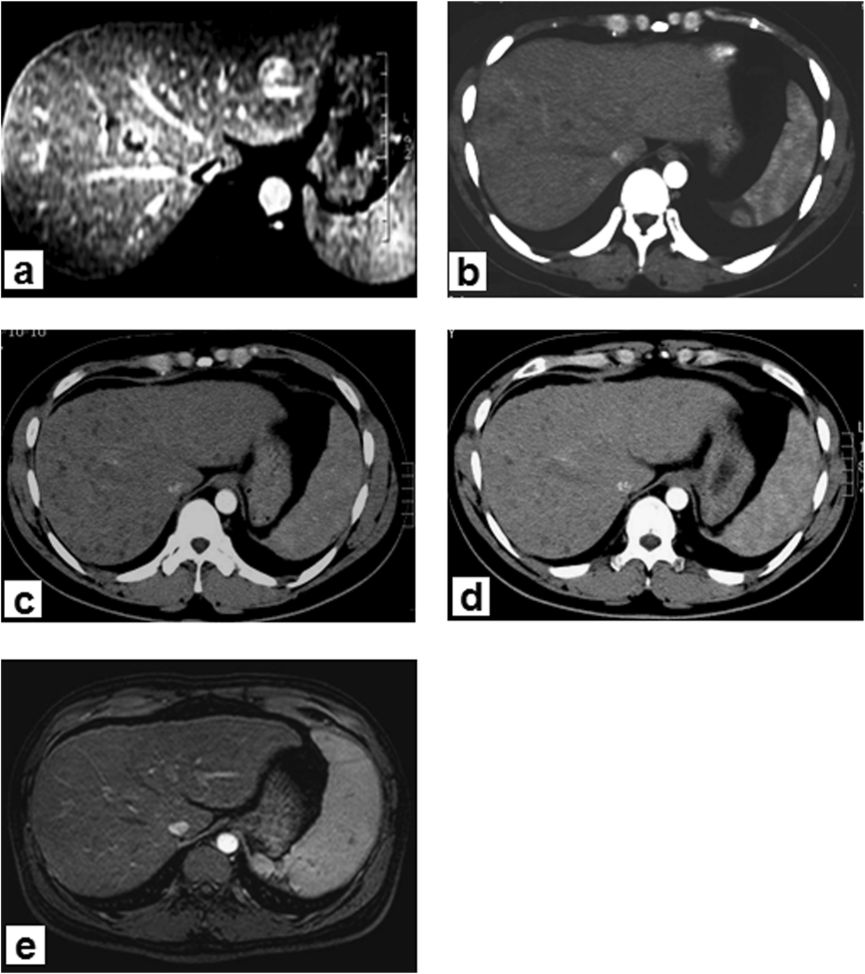
**Abdominal computed tomography (CT) or magnetic resonance imaging (MRI). (a)** Abdominal dynamic enhanced MRI obtained before treatment shows multiple liver lesions, the number of nodules >100, the largest diameter was about 1.55 cm; AFP level, 4,773 ng/ml. **(b)** CT scan obtained 2 months after the start of treatment showed that the lesion was not enhanced in the arterial phase; AFP level, 3.79 ng/ml. **(c)** CT scan obtained 15 months after the start of treatment showed that the number of low-density lesions was less than before, also no enhancement; AFP level, 3.63 ng/ml. **(d)** CT scan obtained 21 months after the start of treatment showed that the diffuse liver lesions disappeared, no abnormal enhancement lesions; AFP level, 2.26 ng/ml. **(e)** MRI scan obtained 46 months after the second treatment showed that there were still no lesions in the liver; AFP, level 3.73 ng/ml.

**Figure 2:**
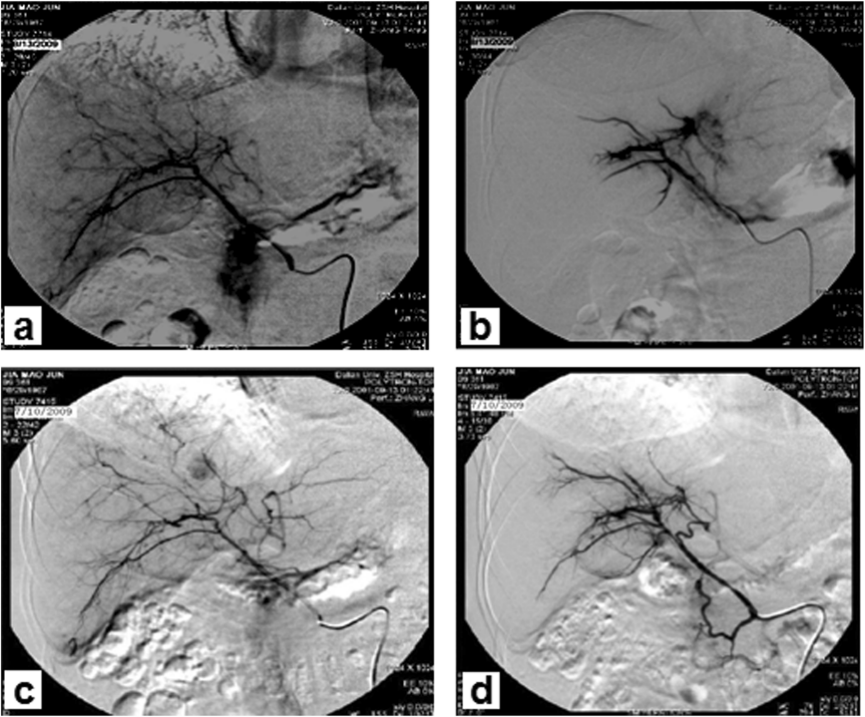
**Hepatic artery angiography. (a)** Hepatic arteriogram shows infiltrative tumor staining in the whole hepatic lobe. Transarterial chemoembolization was performed with a mixture of 30 mg GSPs and 10 mg pirarubicin. **(b)** After GSP embolization, digital subtraction angiography showed that the feeding artery of the tumor was completely blocked. **(c)** 1 month later, a follow-up angiogram shows almost complete disappearance of tumor vascularity. **(d)** A second and final GSP-TACE was performed. After that, the feeding artery of the tumor was completely blocked again.

**Figure 3:**
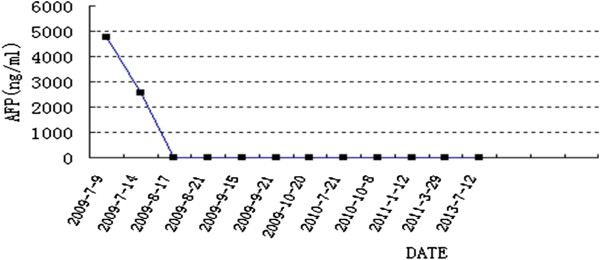
**Change in AFP levels.** When disease was found, the AFP level was 4,773 ng/ml. After the first treatment, the AFP level decreased to 34.4 ng/ml. The AFP level began to drop to normal after the second TACE. Since then, AFP levels have been within the normal range, as reviewed every 1 to 2 months.

A 22-year old male patient presented at our hospital with a 3-month history of acute onset of abdominal pain in July 2009. Contrast-enhanced abdominal magnetic resonance imaging (MRI) revealed more than 100 liver lesions, with the largest having a diameter of about 1.55 cm (Figure 1a). Laboratory results revealed a total bilirubin level of 26 μmol/l, albumin level of 51 g/l, alanine transaminase level of 38 IU/l and α-fetoprotein (AFP) level of 4,773 ng/ml. The patient had a history of chronic hepatitis B infection for 4 years. The clinical diagnosis was hepatocellular carcinoma (diffuse-type). The status of the patient was graded as Child-Pugh class A, with an Eastern Cooperative Oncology Group (ECOG) Performance Status of 0, and was classified by the Barcelona Clinic Liver Cancer (BCLC) system as stage B. Curative treatments, such as surgery or radiofrequency ablation, were not possible, owing to the extent of the disease. Hence, TACE was performed with 30 mg GSPs (350 to 560 μm in diameter) mixed with chemotherapeutic agent pirarubicin, 10 mg). As the nodules were scattered throughout the liver, we performed complete embolization of both hepatic arteries (Figure 2b). One month later, contrast-enhanced abdominal computed tomography (CT) showed significant necrosis of the tumor, mostly without any enhancement in the arterial phase. The patient’s AFP level decreased to 34.4 ng/ml. Once again, TACE was performed with a supersolution of 30 mg GSPs and 10 mg pirarubicin. One month later, contrast-enhanced CT showed no enhancement in the arterial phase (Figure 1b). The patient’s AFP level was 3.79 ng/ml. Since then, AFP levels obtained from testing every one to two months, have been within the normal range (Figure 3). In addition, contrast-enhanced CT was also performed every 3 to 6 months, and showed that the lesions gradually reduced or disappeared with no new local tumor formation. During this period, the patient has been taking antiviral drugs (Lamivudine, 100 mg once daily). At July 2013, the patient had been in complete remission for 46 months according to the modified Response Evaluation Criteria in Solid Tumors (RECIST).

## Discussion

In China, HCC is rare in young adults and mostly occurs in men. In particular, diffuse-type HCC, when presented by young adults, has a typical clinical manifestation that includes high malignancy, lack of early diagnosis and poor prognosis [10, 12]. In accordance with the BCLC clinical staging and treatment system [13], surgical resection is considered to be the primary treatment method. It is more suitable for patients with a single lesion of diameter less than 5 cm, and normal total bilirubin levels without portal hypertension. Radiofrequency ablation is more limited to <3 localized lesions, with tumors ≤3 cm in diameter [14]. Liver transplantation is also considered as one of the curative methods; especially applicable to the patients who are not eligible for the abovementioned methods or where there is no improvement in the patient’s condition [15]. It is also limited to localized lesions, single tumors with diameter less than 5 cm or multiple tumors numbering less than three tumors in total with diameters ≤3 cm, according to Milan criteria [16]. Transcatheter arterial chemoembolization with lipiodol (lipiodol-TACE) is the standard treatment for unresectable intermediate-stage HCC. However, different studies have shown an overall response rate of 17% to 61.9% and a complete tumor necrosis rate of only 0 to 4.8% [17–19].

The DC bead is an embolic drug-eluting bead that is used in the treatment of patients with both primary and secondary liver cancers [20]. Compared with lipiodol-TACE, using this embolization agent to perform TACE can gain higher tumor necrosis and lower postoperative complications [21]. However, DC beads are not available in China. This patient’s cancer was not suitable for the use of permanent embolic agents, such as polyvinyl acetate or DC beads, as these require superselective embolization. The size of the lesions in this patient was <3 cm, and the lesions involved the whole liver. Transcatheter arterial infusion is the only suitable option for such patients. However, we chose GSPs as an alternative particulate embolic agent. The particle diameter is more suitable for use in blood vessels, and better suits the objective of delivering an embolic agent to block tumor target vessels regionally. As GSPs are absorbable particles that dissolve over time, the blocked artery can recanalize in about 2 weeks [22], ensuring the protection of postoperative liver function. In our recent study, we use GSPs to deal with a massive type of HCC, leading to a good efficacy and safety [23].

## Conclusions

In this case, the patient was a young 22-year-old man in whom liver MRI suggested multiple grain-size lesions (>100 nodules). Obviously, he was not a candidate for surgical resection and radiofrequency ablation, nor was liver transplantation indicated, according to the Milan criteria. However, how to acquire effective intervention and long-term survival is a clinical problem. We performed TACE with 350 to 560 μm GSPs mixed with pirarubicin. There were no signs of acute liver failure and the liver function returned to normal after 7 days. The AFP level decreased to normal after the second TACE procedure. To date, the patient has been in complete remission for 46 months. The young man has just married, living a beatific life.

## Consent

Written informed consent was obtained from the patient for publication of this case report and any accompanying images. A copy of the written consent is available for review by the editor-in-chief of this journal.

## Abbreviations

AFP: α-fetoprotein
BCLC: Barcelona Clinic Liver Cancer
CT: computed tomography
ECOG: Eastern Cooperative Oncology Group
GSP: gelatin sponge particle
HCC: hepatocellular carcinoma
MRI: magnetic resonance imaging
RECIST: Response Evaluation Criteria in Solid Tumors (RECIST)
TACE: transcatheter arterial chemoembolization.
